# USABILITY OF A MOBILE APP TO IMPROVE GOAL-DIRECTED BEHAVIOR: CHANGING APATHY INTO ACTION IN PERSONS WITH DEMENTIA

**DOI:** 10.1093/geroni/igz038.107

**Published:** 2019-11-08

**Authors:** Lauren M Massimo, Alexander Miller, Daniel Schiffrin, Sean Lydon, Dawn Mechanic-Hamilton

**Affiliations:** 1 University of Pennsylvania, Perelman School of Medicine, Philadelphia, Pennsylvania, United States; 2 University of Pennsylvania, Department of Neurology, Philadelphia, Pennsylvania, United States

## Abstract

Impairment of goal-directed behavior, often labeled apathy, is the most common and disabling of the behavioral and psychological symptoms of dementia. Activi-daily is a mobile app that targets key and distinct components of goal-directed behavior (initiation, planning and motivation) found to contribute to apathy in persons with dementia. This presentation will focus on usability testing of Activi-daily in persons with dementia and their caregivers. After 1 week of at-home use, we gathered feedback via focus groups on features and usability of Activi-daily. Usability of Activi-daily and its’ potential as an intervention for treating apathy were viewed favorably. Feedback regarding usability revealed that the app was easy for dyads to use and enhanced the patients’ ability to complete their daily tasks with greater independence. Suggestions to improve app usability included adding a voice to text feature, a progress bar to visualize progression towards daily goals and gamification features to enhance motivation.

